# A randomized double-blinded study assessing the dose-response of ropivacaine with dexmedetomidine for maintenance of labor with epidural analgesia in nulliparous parturients

**DOI:** 10.3389/fphar.2023.1205301

**Published:** 2023-08-10

**Authors:** Yao-Hua Shen, Dan M. Drzymalski, Bin-Xiang Zhu, Su-Feng Lin, Fang-Qin Tu, Bei Shen, Fei Xiao

**Affiliations:** ^1^ Department of Anesthesia, Hangzhou City Linping District Maternal and Child Care Hospital, Hangzhou, China; ^2^ Department of Anesthesiology and Perioperative Medicine, Tufts Medical Center, Boston, MA, United Staes; ^3^ Department of Anesthesia, Jiaxing University Affiliated Women and Children Hospital, Jiaxing Maternity and Child Care Hospital, Jiaxing, China

**Keywords:** dose-response, ropivacaine, labor epidural analgesia, breakthrough pain, nulliparous

## Abstract

**Background:** The combination of ropivacaine and dexmedetomidine has been used as an epidural analgesic for inducing labor. However, there is limited data regarding the administration of epidural analgesia for labor maintenance, hence, this study aimed to determine the optimum concentration through dose-response curves of ropivacaine plus dexmedetomidine, which could be used along with the Programmed Intermittent Epidural Bolus (PIEB) technique.

**Methods:** One hundred parturients were randomized into 4 groups who were administered four different doses of ropivacaine (dexmedetomidine at 0.4 μg mL^−1^): 0.04%, 0.06%, 0.08%, and 0.1%. The primary outcome that was determined included the proportion of patients experiencing breakthrough pain during their 1st stage of labor. Breakthrough pain was described as a visual analog scale [VAS] score of >30 mm, requiring supplemental epidural analgesia after the administration of at least one patient-controlled bolus. The effective concentration of analgesia that was used for labor maintenance in 50% (EC50) and 90% (EC90) of patients were calculated with the help of probit regression. Secondary outcomes included epidural block characteristics, side effects, neonatal outcomes, and patient satisfaction.

**Results:** The results indicated that the proportion of patients without breakthrough pain was 45% (10/22), 55% (12/22), 67% (16/24), and 87% (20/23) for 0.04%, 0.06%, 0.08%, and 0.10% doses of the analgesic that were administered, respectively. The EC50 value was 0.051% (95% confidence interval [CI], 0.011%–0.065%) while the EC90 value was recorded to be 0.117% (95% CI, 0.094%–0.212%). Side effects were similar among groups.

**Conclusion:** A ropivacaine dose of 0.117% can be used as epidural analgesia for maintaining the 1st stage of labor when it was combined with dexmedetomidine (0.4 μg mL^−1^) and the PIEB technique.

**Clinical Trial Register:** https://www.chictr.org.cn/index.aspx, identifier ChiCTR2200059557

## Introduction

Epidural analgesia is regarded as the primary tool for managing labor pains owing to its effectiveness and popularity. Over the past decades, several modifications made in the use and administration of epidural labor analgesia have been investigated to achieve the best possible outcomes in patients. Despite considerable advancements, 25% of the patient still experience breakthrough pain (i.e., pain during labor despite a functional epidural catheter) (Paech et al., 1998; Agaram et al., 2009). The proportion of patients experiencing breakthrough pain may be influenced by the concentration of the local anesthetics and adjuvants, different techniques (e.g., combined-spinal epidural, dural puncture epidural), and the course of labor itself (Hess et al., 2001; Tan et al., 2019).

Several studies have proposed the combined application of the programmed intermittent epidural bolus (PIEB) technique and the administration of analgesia for decreasing the breakthrough pain during labor in comparison to the continuous epidural infusion (Wong et al., 2006; Roofthooft et al., 2020). Therefore, the PIEB method is a generally-accepted technique used for labor analgesia. However, while the method of delivering the local anesthetic is important, the concentrations of the local anesthetic and adjuvants that are used are also crucial determinants of the quality of epidural labor analgesia.

Ropivacaine with dexmedetomidine has been used in experimental trials for managing labor pains (Zhang et al., 2019; Liu et al., 2021; Xiang et al., 2021). Although a few researchers have determined dose-response curves of epidural ropivacaine with dexmedetomidine during the induction of labor (Zhang and Li, 2018; Liu et al., 2021; Xiang et al., 2021), the data regarding the maintenance of labor with epidural analgesia, i.e., the combination of ropivacaine and dexmedetomidine, is limited. Therefore, this study aimed to determine the dose-response relationship between ropivacaine and dexmedetomidine as epidural analgesia for labor maintenance. In addition, experiments were conducted to determine the optimum dose of ropivacaine that can be used with the PIEB technique.

## Methods

### Ethics

All experiments conducted in this study were approved by the Ethical Committee of Hangzhou City Lin-Ping District Maternal and Child Care Hospital, Hangzhou, China, (Chairperson Prof Ying-Hao Zhang) on March 23, 2022, (LLSC-KYKT-2022-0006-A). The clinical trial was registered in the Chinese Clinical Trial Register on May 4, 2022, before patient enrollment (ChiCTR2200059557). The patients enrolled in this study were asked to sign a written informed consent form. Finally, this study was initiated on June 1, 2022, and concluded on January 21, 2023.

### Design

This is a single-center, double-blind, randomized, dose-finding study.

### Patients and setting

We enrolled 100 nulliparous parturients based on the Physical Status II Classification System defined by the American Society of Anesthesiologists. The key inclusion criteria implemented in this study included singleton pregnancies at the gestational age of 37–42 weeks, with regular uterine contractions that occurred every 5 min, and those opting for neuraxial analgesia. The key exclusion criteria used in this study included patients who refused the use or presented contraindications to the use of neuraxial analgesia, had a body mass index (BMI) > 40 kg m^−2^, were allergic to ropivacaine or dexmedetomidine, had pregnancy-related issues (e.g., preeclampsia, gestational hypertension, or gestational diabetes), a history of opioid or other sedative use 4 h before analgesia administration, or known fetal abnormalities. Patients who experienced an unintended dural puncture and required emergency cesarean section delivery were excluded.

### Randomization and blinding

Pregnant women who opted for neuraxial analgesia were randomized into one of four groups based on the concentration of ropivacaine that they would be administered, i.e., 0.04%, 0.06%, 0.08%, and 0.1%. Randomization was achieved using a computationally-generated random number sheet (Microsoft Excel for Mac, Microsoft, Redmond, WA). An assistant anesthesiologist, who was unblinded to the patients’ grouping and not involved in data collection, prepared the epidural solutions (ropivacaine plus dexmedetomidine) and marked the epidural infusion pumps as A, B, C, or D (Apon MC ZZB-IV, Jiangsu Apon Medical Technology Co., Ltd., Nantong, China). All other study-related personnel were blinded to the grouping and infusion pumps.

### Initiation of epidural labor analgesia

After entering the labor room, the patients were monitored using standard monitors (such as non-invasive blood pressure, pulse oximetry, electrocardiography, fetal heart rate, and tocodynamometer). Additionally, the baseline heart rate and systolic blood pressure (SBP) values were recorded as the mean of three independent readings measured between uterine contractions. A 250 mL bolus of lactated Ringer’s solution, pre-warmed to 37°C, was initiated 20 min before placing the epidural catheter.

Epidural analgesia was administered in the left lateral decubitus position by applying the “loss of resistance to saline” technique using an 18-gauge Tuohy needle at the L3-4 vertebral interspace (ultrasound confirmed). A 19-gauge nylon multi-orifice epidural catheter-oriented cephalad was inserted 4–5 cm into the epidural space. Following aspiration, if blood and cerebrospinal fluid were absent in the epidural catheter, a test dose (3 mL) comprising lidocaine (1.5%) and epinephrine (15 μg) was administered. After 3 min, the patients were administered ropivacaine (10 mL bolus of 0.1% v/v concentration) with dexmedetomidine (0.4 μg mL^−1^). Patients, who had a pain score of ≤50% on the visual analog scale (VAS) from baseline within 20 min, were considered eligible to continue in the study. Patients who reported a VAS score of >50% from baseline received an 8 mL bolus of 1% lidocaine to determine whether the position of the epidural catheter was appropriate. If the VAS score remained >50% of baseline after 20 min of the rescued bolus, the procedure was regarded as a failure of epidural catheter placement, and the patient was excluded from this study. Thereafter, the epidural catheter was replaced.

### Maintenance of labor with epidural analgesia

Depending on the solution concentrations used in the study groups, the maintenance of labor with epidural analgesia was achieved using ropivacaine with dexmedetomidine at 0.4 μg mL^−1^. The study solution was delivered using the epidural infusion pump (Apon MC ZZB-IV, Jiangsu Apon Medical Technology Co., Ltd., Nantong, China) that was programmed to administer a PIEB dose of 10 mL at an infusion rate of 500 mL h^−1^, which was initiated 1 h after the first loading bolus.

Patients were allowed 5 mL boluses of patient-controlled epidural analgesia (PCEA) with a 10-min lockout interval. The patient was guided to press the PCEA button if she felt pain. If no pain relief was noted, clinicians administered a 10 mL bolus of 0.25% ropivacaine with 100 μg fentanyl according to the principles in our hospital. If pain persisted despite these boluses, it was considered a displacement of an epidural catheter. Thereafter, the patient could not participate in this study.

### Demographic characteristics and outcome assessment

Maternal demographic data, including age, weight, height, blood pressure, and gestational age were recorded. Obstetric characteristics, including cervical dilation at the time of neuraxial analgesia request, and duration of the first and the second stages of labor were recorded.

The primary outcome of this study included the proportion of patients with breakthrough pain in the first stage of labor. Breakthrough pain was defined as a VAS >30 mm within 10 min after administering supplemental epidural analgesia and occurring after the administration of at least one PCEA bolus (Roofthooft et al., 2020).

The secondary outcomes included in this study were the epidural block characteristics (sensory level and motor block), side effects (i.e., hypotension, nausea, vomiting, and Epidural Related Maternal Fever [ERMF] with a temperature of >38°C), neonatal outcomes (i.e., 1- and 5-min Apgar scores, neonatal weight, and umbilical arterial pH), and patient satisfaction.

The sensory block level was assessed using alcohol wipes by asking the patients whether they could sense the temperature, whereas motor block was assessed with the Bromage scale (Bromage, 1965) (0 = able to move the joints in the lower extremity, 1 = can bend the knees and ankles, 2 = can only move the ankles, and 3 = cannot move any of the lower extremity joints). The blood pressure and heart rate values were monitored at 20-min intervals. Maternal hypotension (described as the decrease in SBP to <100 mm Hg or <80% baseline) was treated using rapid intravenous infusion in the left lateral position. If hypotension persisted after 3 min, 20-40-μg phenylephrine bolus was intravenously administered and repeated as needed. The satisfaction levels of the patient were assessed after delivery with the help of a verbal numeric rating scale that ranged between 1 (completely dissatisfied) and 5 (completely satisfied). The uterine contractions and fetal heart rate were continuously monitored with a fetal monitor (FM 20, Philips Medizin Systeme Boeblingen GmbH, Boeblingen, Germany).

The duration of the study ranged from the administration of the initial dose of epidural analgesia to the time of complete cervical dilation. The epidural infusion pump was checked at 60 min intervals by another anesthesiologist who managed the patient’s labor pain to record the number of PCEA boluses that were used.

### Statistical analysis

The sample size was derived using the PASS software (ver. 11.0.7; NCSS, LLC, Kaysville, UT, United States). Calculations based on preliminary data showed that the proportions of patients without breakthrough pain were 0.45, 0.60, 0.75, and 0.90 receiving ropivacaine doses of 0.04%, 0.06%, 0.08%, and 0.1%, respectively. A sample size containing 76 individuals (19 in every group) was required to achieve 90% power for detecting a linear trend by means of a 2-sided Z test and a statistical significance value of 0.05. To allow for probable dropouts and to increase the power of the study, the sample size in this study was randomly increased to 25 individuals in every group (N = 100 in 4 groups). This would also enable us to take into consideration the probability that the true proportion of patients without breakthrough pain differed from the preliminary results noted in this study.

The data were statistically analyzed with the help of the Statistical Package for Social Sciences (SPSS) software for Windows ver. 22.0 (IBM Corp, Armonk, NY, United States) and GraphPad Prism ver. 5.0 (GraphPad Software Inc., San Diego, CA, United States). Continuous data were tested for normality using the Kolmogorov–Smirnov test. The normally-distributed data (which included the duration of 1st and 2nd stages of labor, and neonatal weight) were presented as mean ± standard deviation (SD) and analyzed among different groups using the one-way analysis of variance (ANOVA) (or one-way ANOVA test for trendr) technique. *Post-hoc* Bonferroni pairwise comparisons were then performed to achieve significant results. Furthermore, the non-normally distributed data (patient satisfaction, highest sensory block level, Apgar score, and umbilical artery pH) were presented as the median and interquartile range (IQR) and assessed by means of the Kruskal–Wallis test, where Dunn’s tests were used for *post hoc* pairwise comparison. The chi-square test for trend and the Yates correction was used for assessing the categorical data when the expected number in one or more cells was ≤5.

The effective concentration for maintenance labor with epidural analgesia in 50% of the parturients (EC50) and 90% of the parturients (EC90) was calculated with the probit regression. A 2-sided *p-*value < 0.05 indicated the statistical significance level.

## Results

### Demographic and labor characteristics

**FIGURE 1:**
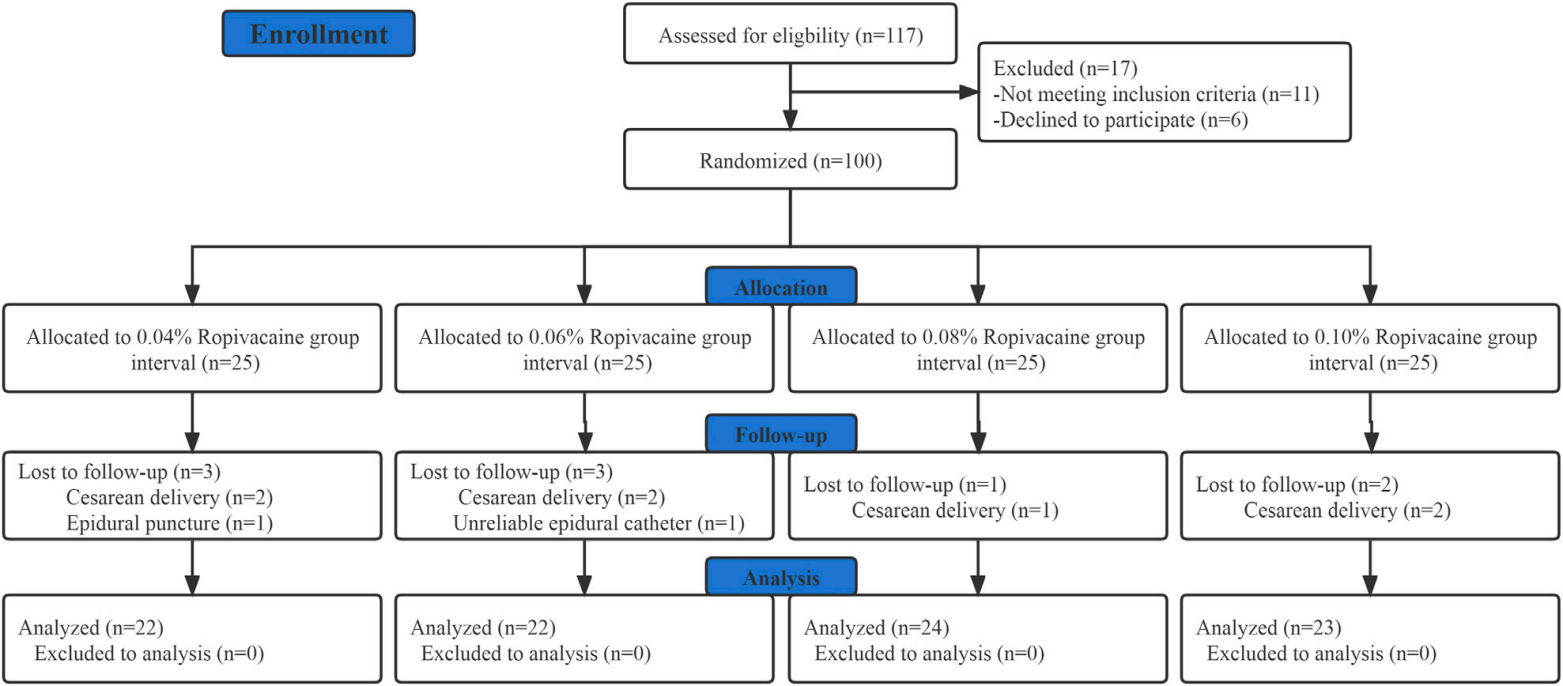
CONSORT flow diagram. *CONSORT, Consolidated Standards of Reporting Trials.

Out of the 117 parturients who were initially screened for eligibility; 11 did not fulfill the inclusion criteria while 6 did not provide their informed consent. Hence, a total of 100 participants were recruited and randomized. Out of these 100 individuals, 9 patients were excluded from the final analysis as shown in Figure 1, of which 7 required intrapartum cesarean delivery, 1 had unintended dural puncture, and 1 had unsuccessful epidural analgesia. Table 1 presents the demographic and labor characteristics of all the selected participants.

**TABLE 1:** Demographics and labor characteristics.

	Randomized concentration of ropivacaine				
Characteristic	0.04% (n = 22)	0.06% (n = 22)	0.08% (n = 24)	0.10% (n = 23)	*p*-value
Age (year)	26.6 ± 3.8	26.2 ± 2.4	26.8 ± 3.2	26.4 ± 3.5	0.25
Height (cm)	159.8 ± 5.7	159.9 ± 4.9	162.3 ± 4.1	160.7 ± 4.2	0.14
Weight (kg)	65.7 ± 6.6	67.9 ± 7.4	66.7 ± 7.2	68.6 ± 5.4	0.701
Gestational age (week)	38.8 ± 1.3	38.7 ± 1.4	39.3 ± 1.4	38.9 ± 1.3	0.431
Cervical dilation at request for epidural analgesia (cm)	1.0 (1.0–2.0)	1.0 (1.0–2.0)	1.0 (1.0–2.0)	2.0 (1.0–2.0)	0.487
Pain score at request for epidural analgesia	8.0 (7.0–10.0)	8.0 (6.8–8.3)	7.0 (6.0–8.8)	8.0 (6.0–9.0)	0.524
Intravenous oxytocin	11 (50)	10 (45)	9 (38)	10 (43)	0.860
Duration of the first stage of labor (min)	592.3 ± 256.1	580.6 ± 320.5	597.6 ± 280.6	544.5 ± 262.5	0.918
Duration of the second stage of labor (min)	52.0 (34.8–68.5)	59.0 (33.8–85.3)	55.0 (33.0–99.5)	66.0 (45.0–101.0)	0.625

Data shown as median (quartiles), or mean (SD) as appropriate.

### Primary outcome and dose-response of epidural ropivacaine

**FIGURE 2:**
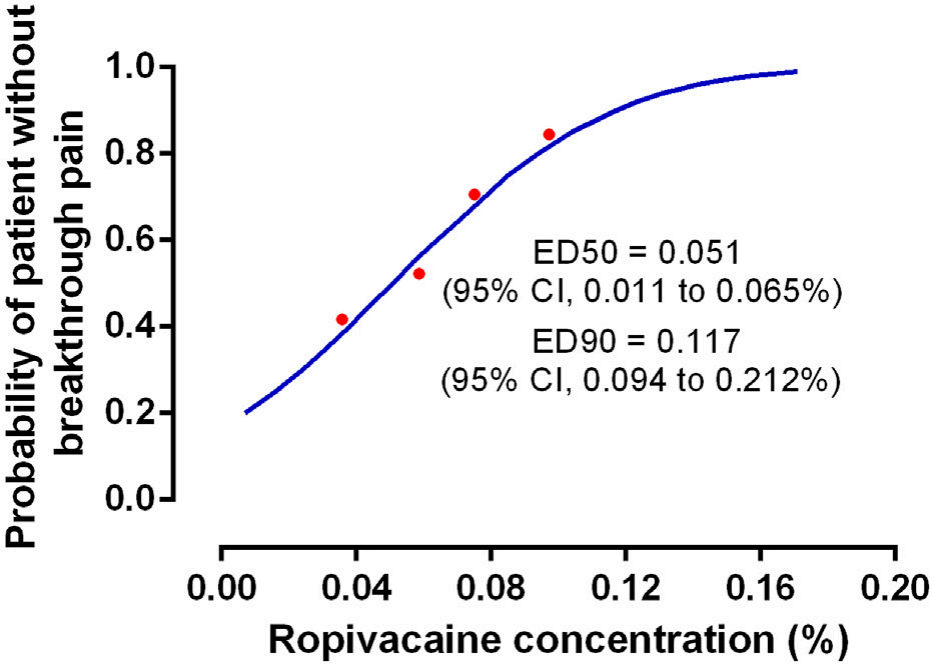
The dose-response curve of the probability of patient without breakthrough pain *versus* ropivacaine concentration. The values of EC50 and EC90 derived from probit analysis were 0.051% (95%CI, 0.011%–0.065%) and 0.117% (95%CI, 0.094%–0.212%), respectively.

The findings indicated that 55% (12/22), 45% (10/22), 33% (8/24), and 13% (3/23) of the patients who received ropivacaine doses of 0.04%, 0.06%, 0.08%, and 0.10%, respectively, displayed breakthrough pain (Table 2). A significant trend (*p* = 0.003) was observed that highlighted the relationship between the ropivacaine dose and the incidence of breakthrough pain. Figure 2 presents the dose-response curve that presents the probability of no breakthrough pain and its relation to the ropivacaine dosage. The EC50 value was recorded to be 0.051% (95% confidence interval [CI], 0.011%–0.065%), whereas the EC90 value was 0.117% (95% CI, 0.094%–0.212%). The Pearson goodness-of-fit chi-square test showed a satisfactory fit of the probit model (*p* = 0.702).

**TABLE 2:** Breakthrough pain, patient satisfaction, and sensory and motor block.

	Randomized concentration of ropivacaine				
	0.04% (n = 22)	0.06% (n = 22)	0.08% (n = 24)	0.10% (n = 23)	*p*-value
Patient suffered Breakthrough pain at least once	12 (55)^a^	10 (45)	8 (33)	3 (13)	0.003
Rate of breakthrough pain (episodes per hour)	0.2 (0.0–0.3)^b^	0.10 (0.0–0.3)	0.00 (0.00–0.1)	0.0 (0.0–0.0)	0.000
Patient Satisfaction	4.0 (3-5)^c^	4.5 (3-5)^d^	5.0 (4-5)	5.0 (5-5)	0.013
Bromage score (0/1/2/3)	22/0/0/0	20/2/0/0	23/1/0/0	20/3/0/0	0.148
Sensory block level	T9.5 (8.0–10.0)	T9.0 (8.0–10.0)	T9.0 (8.0–10.0)	T9.0 (8.0–10.0)	0.635

Data shown as number (%), median (quartiles), or mean (SD) as appropriate.

^a^ Adjust *p* = 0.003 *versus* group 0.10.

^b^ Adjust *p* = 0.022 *versus* group 0.08, adjust *p =* 0.000 *versus* group 0.10.

^c^ Adjust *p* = 0.017 *versus* group 0.10.

^d^ adjust *p =* 0.035 *versus* group 0.10.

### Secondary outcomes

Tables 2, 3 present a summary of the secondary outcomes. All patients exhibited a sensory level > T10 dermatome 20 min after the first epidural bolus. No patient reported a VAS score of >50% from baseline within 20 min of the loading dose. The patients in the different dose groups showed significant differences in the breakthrough pain episodes occurring every hour. The rate of breakthrough pain was more frequent in the patients who received 0.04% ropivacaine compared to those receiving 0.08% and 0.10% doses (adjusted *p =* 0.022 and <0.001). No significant difference was observed in the Bromage score and sensory block levels across different groups (*p* = 0.148 and *p* = 0.635, respectively). Patients who received 0.10% ropivacaine dose exhibited a higher patient satisfaction level compared to those receiving 0.04% and 0.06% doses.

**TABLE 3:** Side effects and neonatal outcomes.

	Randomized concentration of ropivacaine				
	0.04% (n = 22)	0.06% (n = 22)	0.08% (n = 24)	0.10% (n = 23)	*p*-value
Hypotension	2 (9)	1 (5)	2 (8)	2 (9)	0.914
Maternal fever	1 (5)	0 (0)	0 (0)	1 (4)	0.989
1-min Apgar score	10 (9–10)	10 (9–10)	10 (9–10)	10 (9–10)	0.619
5-min Apgar score	10 (10–10)	10 (10–10)	10 (10–10)	10 (10–10)	0.855
Umbilical arterial pH	7.24 (7.21–7.33)	7.28 (7.24–7.29)	7.28 (7.25–7.30)	7.25 (7.24–7.28)	0.379
Neonatal weight, g	3220.9 ± 312.0	3269.1 ± 437.2	3348.6 ± 214.5	3340.3 ± 297.7	0.495
Fetal bradycardia	3 (14)	0 (0)	3 (13)	3 (13)	0.729

Data shown as number (%), median (quartiles), or mean (SD) as appropriate.

### Side effects

The patients across all the groups presented similar side effects (Table 2). They showed a low incidence of hypotension, and none of the patients required vasopressor treatment. One patient in each of the groups, who received 0.04% and 0.10% doses, respectively, experienced ERMF. However, none of the patients across these groups showed any significant difference.

### Neonatal outcomes

Neonatal outcomes, including umbilical arterial pH, 1- and 5-min Apgar scores, and fetal bradycardia have been presented in Table 2. The patients across various groups showed no significant differences in the above neonatal outcomes.

## Discussion

In this study, the EC50 of ropivacaine with 0.4 μg mL^−1^ dexmedetomidine was 0.051% (95% CI, 0.011%–0.065%), which caused no breakthrough pain during maintenance with epidural analgesia at the first stage of labor. The EC90 was 0.117% (95% CI, 0.094%–0.212%). An earlier study observed that the optimum dose of dexmedetomidine that could be used for epidural labor analgesia was 0.4 μg mL^−1^, hence, the same dose has been used in this study (Liu et al., 2021).

Our study is important since it validates the results presented in the earlier studies. In the past, Liu et al. (2021) and Zhang and Li (2018) determined the EC50 value of epidural ropivacaine and dexmedetomidine as 0.062% and 0.044%, respectively, during labor induction. However, because pain increases during labor, the pharmacokinetics may differ during labor compared to induction. Therefore, the results presented in this study can significantly contribute to the literature because we investigated the EC50 of ropivacaine with dexmedetomidine for maintaining labor with epidural analgesia. Furthermore, the EC90 results presented in this study exhibit a higher clinical relevance.

This study also defines breakthrough pain. In direct contrast to other studies (Sng et al., 2015; Zuo et al., 2022), breakthrough pain is defined as a VAS score of >30 mm, where a supplemental epidural bolus is needed after the patients have been administered PCEA bolus during the maintenance of the first stage of labor. We believe that this approach is clinically relevant compared to the past studies (Epsztein Kanczuk et al., 2017; Bittencourt et al., 2019; Song et al., 2022; Yao et al., 2022), which define effective labor analgesia as requiring no PCEA bolus for maintenance of labor. Considering that all parturients were allowed PCEA, it is important to study the pharmacokinetics in a clinical context. It is believed that further investigation into the clinically-relevant scenarios can lead to fewer undesirable side effects, such as excessive motor block, high sensory block, and severe hypotension. Furthermore, the definition of breakthrough pain used in this study allows the patient with at least some control over her pain, which is an important determinant of patient satisfaction (Merrer et al., 2021). However, none of the earlier studies have compared the effects of different definitions of breakthrough pain in clinical management. Hence, the studies that have focused on this topic need further validation.

Although the findings of this study suggested that 0.117% was the optimum concentration of ropivacaine that could be combined with 0.4 μg mL^−1^ dexmedetomidine for labor maintenance with epidural analgesia, it is important to remember that these results can be applied under the conditions used in this study. Factors affecting the results include differences in study protocols, statistical analyses, definitions of breakthrough pain, adjuvant drugs, doses of local analgesia, the use of epidural test doses, and the extent of cervical dilatation. Thus, we suggest that the use of epidural analgesia for labor management should be implemented according to the patient’s response to medication for providing a flexible regimen rather than a fixed one. Differences in the risk of hypotension, maternal fever, and breakthrough pain, indicate that it is important to provide individualized care for parturients opting for analgesia during labor.

Our study has several limitations that should be considered before implementing the results in clinical practice. Firstly, the generalizability of the results is limited due to strict inclusion criteria; hence, the results would vary in patients with obesity, shoulder dystocia, or other comorbidities or clinical factors. Secondly, as the EC90 value was outside the range of the study groups, further studies need to be performed using higher concentrations compared to those tested in this study. Thirdly, because the observational period was restricted to the first stage of labor, these results cannot be extrapolated to the 2nd stage of labor. Higher epidural analgesia doses may be needed for patients who are in the 2nd stage of labor. Fourth, because the PIEB interval was set to 60 min in this study, the EC50 and EC90 values might vary with the time interval. Further, we did not observe a direct clinical analgesic response at the EC90 value because it was outside the selected dose range. Thus, further studies are needed that focus on the EC90 value of epidural ropivacaine.

To summarize, based on the findings of this study and after considering the study scenario, a ropivacaine dose of 0.117% has been recommended in this study for epidural analgesia when combined with dexmedetomidine 0.4 μg mL^−1^ and a 60-min PIEB technique during the first stage of labor.

## Data Availability

The original contributions presented in the study are included in the article/supplementary material, further inquiries can be directed to the corresponding authors.
